# A systematic review and meta-analysis of the impact of tuberculosis on health-related quality of life

**DOI:** 10.1007/s11136-012-0329-x

**Published:** 2012-12-12

**Authors:** M. Bauer, A. Leavens, K. Schwartzman

**Affiliations:** 1 Respiratory Epidemiology and Clinical Research Unit, Montreal Chest Institute, McGill University Health Centre, Room K1.28, 3650 St. Urbain, Montreal, QC H2X 2P4 Canada; 2 Respiratory Epidemiology and Clinical Research Unit, Montreal Chest Institute, McGill University Health Centre, Room K1.23, 3650 St. Urbain, Montreal, QC H2X 2P4 Canada

**Keywords:** Tuberculosis, Health-related quality of life, Systematic review, Meta-analysis

## Abstract

**Purpose:**

To summarize the impact of tuberculosis (TB) on quantitative measures on self-reported health-related quality of life (HRQOL).

**Methods:**

We searched eight databases to retrieve all peer-reviewed publications reporting original HRQOL data for persons with TB. All retrieved abstracts were considered for full-text review if HRQOL was quantitatively assessed among subjects with TB. Full-text articles were reviewed by two independent reviewers using a standardized abstraction form to collect data on socio-demographic characteristics, questionnaire administration, and mean HRQOL scores. Meta-analyses were performed for standardized mean differences in HRQOL scores, comparing subjects treated for active TB with subjects treated for latent TB infection (LTBI), or with healthy controls, at similar time points with respect to diagnosis and/or treatment.

**Results:**

From over 15,000 abstracts retrieved, 76 full-text articles were reviewed, which represented 28 unique cohorts (6,028 subjects) reporting HRQOL among subjects with active TB; 42 % were women and mean age was 42 years. Data on key social and behavioral determinants were limited. Within individual studies and in meta-analyses, subjects with active TB disease consistently reported worse HRQOL than concurrently evaluated subjects treated for LTBI. However, meaningful improvements in HRQOL throughout active TB treatment were reported by longitudinal studies.

**Conclusions:**

In a variety of studies, in different settings and using different instruments, subjects with active TB consistently reported poorer HRQOL than persons treated for LTBI. Future research on HRQOL and TB should better address social and behavioral health determinants which may also affect HRQOL.

## Introduction

One-third of the world’s population is infected with *Mycobacterium tuberculosis*, which causes the infectious respiratory disease tuberculosis (TB) [34]. In 2010, the World Health Organization (WHO) estimated 8.8 million new cases of TB, and 1.1 million deaths from TB among HIV-negative individuals [11, 54].

Active TB disease exerts a substantial toll on quality of life—ranging from somatic symptoms related to disease and treatment to psychological distress from social isolation and stigmatization [18, 25, 47]. The diagnosis of latent TB infection (LTBI) may be misconstrued as active TB disease or even HIV infection, both of which may also lead to further stigmatization; in some communities, the diagnosis of TB is strongly associated with HIV infection [36, 50, 58]. Health-related quality of life (HRQOL), as reported by patients, is therefore highly relevant to understanding and quantifying the true impact of TB.

The purpose of this research is to summarize the impact of TB on quantitative measures of self-reported HRQOL. We have conducted a systematic review of self-reported HRQOL among persons with active TB, LTBI, and those with persistent pulmonary symptoms following treatment of active TB. This updates and supplements a previous systematic review, which addressed HRQOL among TB patients, by expanding the search strategy and searching more databases [22].

## Methods

### Identification and selection of relevant publications and research

A librarian trained in systematic reviews of medical literature was consulted to construct a comprehensive search strategy. Terms included in the search strategy related to the concepts of the operational definition of HRQOL as described by Wilson and Cleary [55]. We searched 8 databases to retrieve relevant peer-reviewed publications, reporting original research: PubMed, EMBASE, EMBASE classic, PsycINFO, HaPI, BIOSIS, The Cochrane Library, and CINAHL. Databases were searched for articles published between January 1, 1960 and April 1, 2011. The search strategy is provided in “Appendix 1”.

All retrieved abstracts were exported to EndNote X4 software and screened by one author (MB); any abstract that reported self-rated, quantitative measures of HRQOL among TB subjects was eligible for full review. Two authors, fluent in English, read full-text versions of all articles written in English and completed a standardized abstraction form for all studies that evaluated HRQOL among individuals with active TB, LTBI, or post-TB pulmonary sequelae. Abstracted information was reviewed and discrepancies discussed between these two reviewers. If a discrepancy arose, the two authors reviewed the original article together to reach consensus. For those articles published in a language other than English, other research personnel fluent in the language of the publication, as well as English, completed the abstraction form. References cited among the included publications were scanned for additional potential relevant studies, which were also reviewed, if eligible, using the abstraction form.

Articles were excluded from the review if (1) quantitative measures of HRQOL were not available or if there were no subjects with TB included, (2) if data for subjects with and without TB were aggregated together, (3) if the full-text articles were not accessible to reviewers, or (4) if the publication was written in a language that the reviewers were unable to understand. Studies using the standard gamble instrument were included, since it provides an assessment of health utility, a quantitative measure of HRQOL that incorporates uncertainty, which is particularly relevant to health care decision makers.

### Data extraction

The standardized abstraction form captured the following information:

- socio-demographic characteristics of subjects
- clinical characteristics for subjects with active TB (pulmonary/extra-pulmonary disease, smear status, re-treatment)
- behavioral risks (smoking, alcohol abuse, and injection drug use),
- study design features (subject recruitment mechanisms and inclusion/exclusion criteria),
- accounting for subjects who were (a) eligible from the target population, (b) approached of those eligible, (c) recruited of those approached, (d) completed evaluations of those recruited, and (e) included in the analysis of those who completed evaluations,
- HRQOL questionnaire administration (subject self-administered/interviewer-administered, language of administration, timing with respect to TB diagnosis and treatment, proxy respondents), and
- HRQOL results, by subject group

The abstraction form also included a quality rating score using a 3-point scale (2 being well-described, 1 poorly described, and 0 not described in article) for each of the following eight attributes: study population, sampling mechanism, accounting for potential subjects not included in the analyses, quality check of responses, explicit description of HRQOL instruments, data entry check, training of interviewers, and discussion of study strengths and limitations. These eight items were extracted from the *STROBE Statement*—*checklist of items that should be included in observational studies (version 4)* based on study characteristics anticipated to vary widely across studies, with particular focus on methods and results [48]. Summary quality ratings were calculated by summing the scores for each of these eight categories; the minimum and maximum possible scores were 0 and 16, respectively.

### Statistical analysis

Data extracted from original publications were recorded in Microsoft Excel (2010). Demographics, disease characteristics, and HRQOL measures were extracted and summarized by sub-group (active TB, LTBI, TB-free controls, etc.) Variables describing study design and methods were summarized.

We used Microsoft Excel (2010) to calculate standardized mean differences of HRQOL scores between the group of subjects treated for active TB in a given study and a concurrently evaluated comparison group. Meta-analyses were performed for standardized mean differences among studies that compared similar groups and administered the HRQOL questionnaires at similar time points with respect to TB diagnosis and/or treatment. For those publications that were deemed eligible for meta-analysis but did not report the particular measure of interest, investigators were contacted to request these data.

For each group of studies included in a given meta-analysis, we used MIX 2.0 Pro to calculate the random effects pooled estimates, using the DerSimonian-Laird method, of standardized mean differences in HRQOL scores [5, 23]. We used MIX 2.0 Pro to produce forest plots and calculate the *I* ^2^ statistics, with 95 % confidence intervals (CI) [5, 23]. These statistics allowed us to assess heterogeneity among studies’ standardized mean differences in HRQOL scores.

We used Microsoft Excel (2010) to calculate effect sizes among studies with longitudinal measures of HRQOL, comparing subsequent with initial measurements [8]. Any effect size of at least 0.50 was considered a meaningful change in HRQOL [43]. We also compared changes in HRQOL scores to previously published estimates of minimum important difference for the relevant instruments, when available.

## Results

**Fig. 1:**
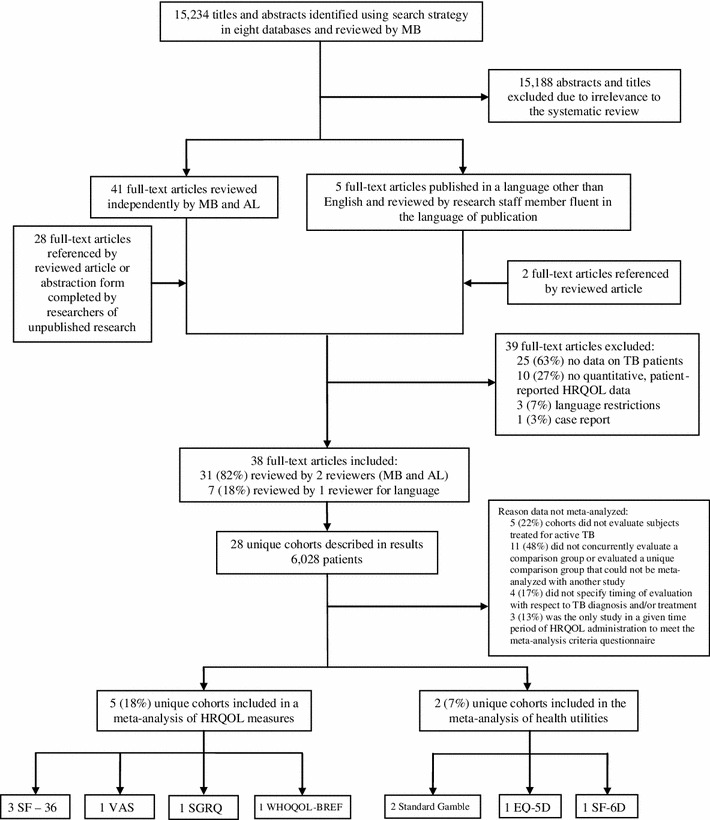
Sampling and selection of published literature on HRQOL among tuberculosis patients from January 1, 1960–April 1, 2011. SF-36 is short form-36, VAS is visual analog scale; SGRQ is St. George’s respiratory questionnaire; WHOQOL-BREF is the World Health Organization’s Quality of Life BREF; EQ-5D is the EuroQoL 5D; SF-6D is the 6-dimension health utility scores derived from 11 items of the SF-36

The search strategy yielded over 15,000 abstracts, 46 of which were eligible for full-text review. We looked for additional publications in the references of each of these 46 articles to retrieve an additional 30 articles for full-text review (Fig. 1).

Of the 76 full-text articles, 38 (representing quantitative HRQOL evaluations among 28 unique cohorts of TB patients) were relevant to our review [1–4, 6, 9, 10, 12–21, 26, 29, 31–33, 35, 37, 38, 39, 40, 45, 46, 49, 51–53, 56, 57, 59, 60]. Studies included in the systematic review were published in English, Korean, Chinese, Spanish, and Turkish. Five unique cohorts contributed to meta-analyses; all articles in the meta-analysis were published in English [4, 15–17, 33, 38]. Five unique cohorts contributed to analysis on meaningful changes in effect sizes of HRQOL measures over time [14, 15, 29, 32, 33].

The 28 cohorts together included 6,028 subjects from 16 countries, across 5 continents. Twenty-one studies (75 %) collected cross-sectional data, and 7 studies (25 %) studies used a longitudinal design. There were no randomized controlled trials conducted among TB patients that included quantitative, patient-reported HRQOL measures. Primary data collection for these studies occurred between 1992 and 2011.

### Characteristics of all subjects

Twenty-seven of the 28 unique cohorts reported the number of women, which corresponded to 42 % of the subjects in these studies [1–4, 6, 9, 10, 12–15, 18–21, 26, 29, 31–33, 35, 37, 38, 39, 40, 45, 46, 49, 51–53, 56, 57, 59, 60]. Mean age (reported in 13 studies) was the most frequently reported summary measure of age [1–4, 6, 13–15, 20, 21, 26, 32, 33, 38, 45, 46, 52, 53, 59, 60]. Although the mean age of subjects across these 13 studies was 42 years, the mean age ranged from 26 to 62 years within individual studies. Among the 5 studies reporting the proportion of foreign-born subjects in their sample, 75 % of all subjects were foreign-born, ranging from 48 to 89 % across these studies [4, 21, 29, 33, 38]. Eight studies stated they excluded known TB/HIV co-infected patients or included these patients and provided sero-status—696 (29 %) of all subjects with known sero-status were co-infected with TB/HIV, which ranged from 0 to 100 % across these studies [3, 4, 12, 16, 17, 26, 32, 33, 38]. Information on subjects’ health behaviors and socioeconomic profiles was extremely limited (Table 1).

**Table 1:** Total sample and sub-group characteristics among subjects of reviewed studies at the time of subjects’ initial HRQOL evaluation [1–4, 6, 9, 10, 12–15, 18–21, 26, 29, 31–33, 35, 37, 38, 39, 40, 45, 46, 49, 51–53, 56, 57, 59, 60]

Subject Group	Characteristics	Studies reporting	Number of subjects with available information (% of subjects in that category)	Number of subjects (%) with attribute
All subjects (*N* = 6,028) 36 articles, 28 unique cohorts of TB patients	Demographics			
	Women	27	5805 (96)	2408 (42)
	Mean age—years	13	2001 (33)	42
	Foreign-born	6	1049 (17)	790 (75)
	Median duration in study country—years	3	368 (6)	3–7
	Racial/ethnic minority	6	991 (16)	517 (52)
	Socioeconomic			
	Completed ≤ primary school % per study	14	3820 (63)	8–89
	Unemployed % per study	9	2316 (39)	7–80
	Co-infection status and health behaviors			
	HIV co-infection	10	2360 (39)	696 (29)
	Smokers	9	1602 (27)	597 (37)
	Alcohol abuse	5	916 (15)	444 (48)
	Injection drug use	2	368 (6)	19 (5)
Active TB (*n* = 3,541, 59 %), 24 unique cohorts	Demographics			
	Women	23	3524 (99)	1360 (39)
	Mean age—years	18	2233 (63)	40
	Foreign-born	5	416 (12)	324 (78)
	Median duration in study country—years	2	122 (3)	3–7
	Racial/ethnic minority	–	–	–
	Socioeconomic			
	Completed ≤ primary school % per study	12	2410 (68)	9–76
	Unemployed % per study	9	1749 (49)	7–80
	Co-infection status and health behaviors			
	HIV co-infection	8	722 (20)	213 (30)
LTBI (*n* = 639, 11 %), 6 unique cohorts	Demographics			
	Women	5	614 (96)	308 (48)
	Mean age—years	5	614 (96)	39
	Foreign-born	3	299 (47)	260 (41)
	Co-infection status and health behaviors			
	HIV co-infection	5	561 (88)	16 (3)
Controls (*n* = 1049, 17 %), 8 unique cohorts	Demographics			
	Women	5	611 (58)	251 (24)
	Mean age—years	5	611 (58)	38
	Socioeconomic			
	Completed ≤ primary school % per study	4	383 (37)	7–26

**Table 2:** Discrimination of HRQOL instruments at initial evaluation by patient groups, among 24 unique studies evaluating persons with active TB [4, 16–27, 38]

Measures of HRQOL instrument discrimination	Patient groups		
	Total sample	Active TB	LTBI
Ceiling effects			
Number of studies reporting	3	2	1
Range of proportion of subjects reporting maximum score per study	5.4–53	0–25	7.2
Floor effects			
Number of studies reporting	2	2	1
Range of proportion of subjects reporting minimum score per study	0−4.0	0–2.9	0

**Table 3:** Quality rating scores of articles comprising the 28 unique cohorts evaluating HRQOL among patients with active TB

Study [Reference]	1	2	3	4	5	6	7	8	Summary score
Aghanwa [1]	2	2	0	0	2	0	1	1	8
Aydin [2]	2	1	0	0	2	0	0	1	6
Babikako [3]	2	1	0	0	2	2	0	2	9
Bauer [4]	2	2	1	2	2	0	2	2	13
Bhatia [6], Dellborg [9, 10], Engstrom [19], Olofson [37]	1	0	0	0	1	0	0	0	2
	1	2	2	0	2	0	0	1	8
Deribew [12]	2	2	2	0	2	0	1	2	11
Dhingra [13, 14]	1	2	1	0	2	0	0	1	7
Dhuria [15]	2	0	1	0	2	0	0	2	7
Dion [16, 17]	2	2	2	0	2	0	0	2	10
Fu [20]	2	0	0	0	0	0	0	0	2
Guo [21]	2	1	2	0	2	0	0	1	8
Husain [26]	1	1	1	0	2	0	1	2	8
Kruijshaar [29]	2	2	2	0	2	0	1	2	11
Lopez-Campos [31]	1	0	0	0	2	0	0	1	4
Maguire [32]	2	0	2	0	1	0	0	2	7
Marra [33]	0	2	2	0	2	0	2	1	9
Muniyandi [35]	1	0	2	2	2	2	2	2	13
Pasipanodya [38]	0	2	2	0	2	2	0	1	9
Pehrsson [39]	1	0	2	0	1	0	0	1	5
Rajeswari [40]	2	1	2	0	2	2	0	1	10
Unalan [45, 46]	2	0	2	2	2	2	0	1	11
Vinaccia [49]	1	0	0	0	2	0	0	1	4
Westaway [53]	1	2	2	0	2	0	2	1	10
Windisch [56, 57]	2	2	1	0	1	0	0	2	8
Yang [59]	2	0	0	0	0	0	0	0	2
Yelken [60]	1	0	1	0	1	0	0	0	3

The eight items in the quality rating tool were extracted from the *STROBE Statement*—*checklist of items that should be included in observational studies (version 4)* based on study characteristics anticipated to vary widely across studies, with particular focus on methods and results [48]

Item 1: description of study population

Item 2: description of sampling mechanism

Item 3: accounting for losses to follow-up

Item 4: quality check of HRQoL responses performed during data collection

Item 5: description of HRQoL instruments used in data collection

Item 6: data entry check before analysis

Item 7: interviewer training (before and throughout data collection process)

Item 8: discussion of strengths and limitations of study

**Table 4:** Effect sizes for longitudinal changes in HRQOL measures reported by subjects treated for active TB

Author [reference]	Instrument^a^	Time1 (T1)	Time2 (T2)	Time3 (T3)	Effect size, T1–T3	Effect size, T1–T2	Effect size, T2–T3
Dhingra [13] Dhingra [14]	DR-12	Start of TB treatment	2 months treatment	End of treatment	2.05^b^	1.72^b^	0.56^b^
Dhuria [15]	WHOQOL-BREF	Start of TB treatment	3 months treatment	End of treatment	1.51^b^	1.03^b^	0.58^b^
Kruijshaar [29]	SF-36—PCS	TB diagnosis	2 months treatment	–	–	0.23	–
Kruijshaar [29]	SF-36—MCS	TB diagnosis	2 months treatment	–	–	0.72^b^	–
Kruijshaar [29]	STAI-6	TB diagnosis	2 months treatment	–	–	0.24	–
Kruijshaar [29]	CES-D	TB diagnosis	2 months treatment	–	–	0.72^b^	–
Maguire [32]	Modified SGRQ	TB diagnosis	2 months treatment	6 months treatment	2.64^b^	1.80^b^	0.97^b^
Marra [33]	SF-36, PCS	TB diagnosis	–	6 months treatment	0.06	–	–
Marra [33]	SF-36, MCS	TB diagnosis	–	6 months treatment	0.19	–	–
Marra [33]	BDI	TB diagnosis	–	6 months treatment	0.31	–	–

^a^WHOQOL-BREF is the World Health Organization’s Quality of Life BREF. SF-36 PCS and MCs are the physical and mental component scores of the Short-Form 36 questionnaire, respectively. STAI-6 is the State-Trait Anxiety Inventory Short Form. CES-D is the Center for Epidemiologic Studies Depression Scale. Modified SGRQ is a modified version of the St. George’s Respiratory Questionnaire. BDI is the Beck Depression Inventory

^b^Meaningful change in HRQOL scores as defined by Cohen’s criteria [8, 43]

Twenty-four of the 28 unique studies (representing 3,541 subjects, 59 % of the total sample) conducted quantitative assessments of HRQOL among persons with active TB [1–4, 6, 12–15, 18, 20, 21, 26, 29, 32, 33, 35, 38, 40, 45, 46, 49, 51–53, 59, 60]. Fourteen of these studies concurrently evaluated HRQOL among other patient groups—6 studies evaluated persons with LTBI, while 8 evaluated TB-free, healthy control subjects [1–3, 15–17, 20, 21, 33, 38, 45, 46, 51–53, 59] (Table 1).

The remaining 4 of the 28 unique studies evaluated HRQOL among subjects with patients with post-TB sequelae who developed chronic alveolar hypoventilation (CAH) and were using home mechanical ventilation (HMV); see “Appendix 4” and Table 7 in Appendix [9, 10, 19, 37, 39, 56, 57].

### Classification of TB patient groups

Fifteen of the 24 studies evaluating HRQOL among patients with active TB specified that disease diagnosis was based on smear and/or culture confirmation, and/or the use of standardized clinical and radiographic criteria (e.g., those of the WHO) [3, 4, 12, 15–18, 26, 29, 32, 33, 38, 45, 46, 49, 51, 52, 60]. Nineteen studies (2,586 subjects with active TB, 73 %) specified the disease site of these subjects—2,350 (91 %) had pulmonary TB; 888 (38 %) of these individuals were sputum smear-positive at their initial HRQOL evaluation, which generally indicates more severe and contagious disease [1–4, 12–15, 20, 26, 29, 32, 33, 35, 38, 49, 51–53, 59, 60].

A total of 639 subjects treated for LTBI (11 % of the total sample) were concurrently evaluated in 6 studies that assessed HRQOL among subjects with active TB [4, 16, 17, 21, 33, 38, 45, 46]. Five of these 6 studies explicitly stated that subjects with LTBI were diagnosed by a positive tuberculin skin test (TST), representing 533 (88 %) of all LTBI subjects [4, 16, 17, 33, 38, 45, 46].

A total of 1049 healthy control subjects (17 % of the total sample) were concurrently evaluated in 8 studies that assessed HRQOL among subjects with active TB [1, 4, 15, 20, 45, 46, 49, 51, 52, 59]. Confirmation of healthy status was described in only 3 studies; diagnostic criteria included a physical exam plus chest radiographs and electrocardiograms, and/or a negative TST result [4, 20, 45, 46].

### HRQOL and health utility instruments

Thirty-four different HRQOL and health utility instruments were used among the studies described in this review (Table 5 in Appendix). The most commonly used tool was the SF-36; 8 of the 28 unique cohorts reported HRQOL measures from the SF-36 [3, 4, 17, 21, 29, 33, 35, 51, 52]. Most studies used only one instrument, but three studies used as many as four tools [16, 17, 29, 39]. Of those studies reporting the method of assessment, 11 used only interviewer-administered questionnaires, 9 studies used only self-administered questionnaires, and 5 used both [1–4, 6, 9, 10, 12, 15–19, 21, 26, 31, 33, 35, 37, 38, 39, 40, 45, 46, 49, 51, 52, 56, 57, 59, 59]. One study permitted proxy respondents [60]. Twenty-one of the 28 studies that evaluated subjects treated for active TB stated that the administered HRQOL instrument was previously validated or that their research was the validation study for this tool [2–4, 6, 9–19, 21, 26, 29, 31, 33, 35, 37, 38, 39, 40, 45, 46, 49, 51–53, 56, 57]. Four of these studies explicitly described ceiling effects, and three explicitly addressed floor effects (Table 2) [4, 16–27, 38].

### Assessment of data quality

The mean quality rating score was 7.3, with scores ranging from 2.0 to 13.0. The mode was 8.0. (The greater the study rating score, the better the perceived quality). Four of the 28 unique cohorts reported a process to check for questionnaire comprehension [4, 16, 17, 35, 45, 46]. However, only one reported the numbers of subjects removed because of poor comprehension [16, 17]. Twelve reported the number of subjects who met the investigators’ inclusion criteria [9, 10, 16, 17, 19, 21, 26, 32, 33, 35, 37, 38, 39, 40, 45, 46, 53]. The proportion of potential subjects who refused participation ranged from 0 to 37 %, while the proportion of subjects who were lost to follow-up ranged from 0 to 8 % (Table 3).

### Meta-analyses

**Fig. 2:**
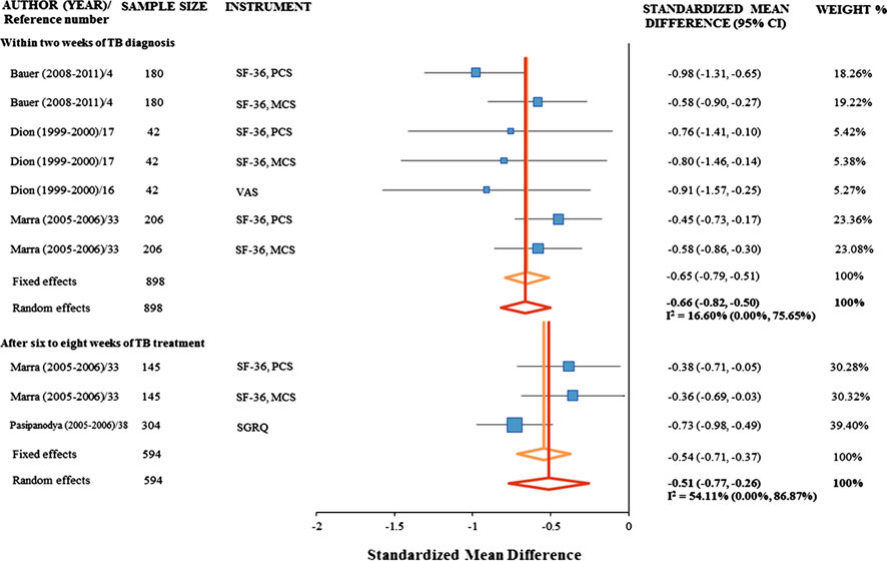
Standardized mean differences between groups of subjects treated for active TB compared to groups of subjects treated for latent TB infection, stratified by timing of HRQOL evaluation with respect to TB diagnosis and treatment

Separate meta-analyses were performed for HRQOL measures and health utility measures. Data from three unique cohorts, using two unique instruments, contributed to the meta-analysis of standardized mean differences in HRQOL between subjects treated for active TB and subjects treated for LTBI within 2 weeks of TB diagnosis [4, 16, 17, 33]. The random effect pooled estimate for the standardized mean difference was −0.66 (95 % CI −0.82, −0.50), and the *I* ^2^ statistic was 17 % (0, 76 %). We then repeated the meta-analysis, excluding the unpublished data of Bauer et al. The random effect pooled estimate for the standardized mean difference in HRQOL scores was −0.58 (95 % CI −0.75, −0.40), and the *I* ^2^ statistic was 0 % (0, 79 %). Data from two cohorts, using two instruments, contributed to the meta-analysis of standardized mean differences in HRQOL between subjects treated for active TB and subjects treated for LTBI after completing 6–8 months of treatment [33, 38]. The random effects estimate for the standardized mean difference was −0.51 (−0.77, −0.26), and the *I* ^2^ statistic was 54 % (0, 87 %) (Fig. 2).

Two cohorts (one unpublished), using two instruments, met our meta-analysis criteria and evaluated subjects treated for active TB compared to healthy controls within 2 weeks of TB diagnosis [4, 15]. The random effects pooled estimate of the standardized mean differences in HRQOL of these two patients groups was −1.14 (−1.75, −0.54), and the *I* ^2^ statistic was 0 % (0, 85 %). In both cohorts, the standardized mean differences indicated lower mean HRQOL scores reported by subjects with active TB disease than by healthy control participants. Findings were more similar for standardized mean differences in SF-36 PCS scores (−1.22, 95 % CI −1.58, −0.85; Bauer et al. [4]) and WHOQOL-BREF scores (−1.62, 95 % CI −1.96, −1.28; Dhuria et al. [15]) than for SF-36 MCS scores (−0.58, 95 % CI −0.93, −0.24; Bauer et al. [4]).

Two cohorts (one unpublished), using two instruments, contributed to the meta-analysis of standardized mean differences in health utilities reported by subjects treated for active TB and subjects treated for LTBI, within 2 weeks of their TB diagnosis [4, 16, 17]. The random effects pooled estimate of the standardized mean differences in health utilities of these two patients groups was −0.62 (−0.82, −0.42), and the *I* ^2^ statistic was 89 % (69, 96 %). The standardized mean differences in Standard Gamble scores (−0.65, 95 % CI −0.96, −0.33) and in SF-6D scores (−0.76, 95 % CI −1.08, −0.44) in the unpublished cohort of Bauer et al. [4] suggested a greater decrement in health utility than previously reported by Dion et al. In the latter study, the standardized mean differences were −0.19 (95 % CI −0.82, 0.45) for Standard Gamble scores [16] and −0.42 (95 % CI −1.06, 0.21) for EQ-5D scores [17].

Further details about HRQOL and health utility scores in these studies are presented in “Appendix 2” and Table 6 in Appendix.

### Meaningful changes in longitudinal HRQOL measures

Five of the 7 unique cohorts using a longitudinal design reported mean HRQOL scores from subjects treated for active TB at at least two time points; one of these five cohorts also reported longitudinal HRQOL scores for subjects treated for LTBI [13–15, 29, 32, 33]. Table 4 displays the calculated effect sizes, comparing later with initial values. Additional information is presented in “Appendix 3”.

The greatest improvement in HRQOL occurred during the first 2–3 months of treatment, among studies that evaluated HRQOL among subjects treated for active TB at the beginning of treatment, after the initial phase of treatment, and at/near the end of treatment [13–15, 32]. Based on the effect sizes, instruments assessing mental well-being also indicated meaningful improvements in HRQOL after 2 months of treatment [29].

Among subjects treated for LTBI, longitudinal measurements of HRQOL did not suggest meaningful changes between diagnosis and 6 months of treatment [33].

## Discussion

Subjects with active TB consistently reported poorer HRQOL than subjects treated for LTBI and untreated controls, across a variety of questionnaires and settings. For example, random effects estimates of pooled standardized mean differences demonstrated that subjects treated for active TB had mean scores 0.66 and 0.51 standard deviations below those treated for LTBI within 2 weeks of diagnosis and after 6–8 months of treatment, respectively. Pooled estimates of standardized mean differences in health utilities among subjects treated for active TB compared to those treated for LTBI within the first 2 weeks of treatment showed similar results. The difference between subjects treated for active TB and healthy controls was even more pronounced. Other studies of HRQOL and health utility measures identified by our systematic review, but which could not be meta-analyzed, also reported a consistently detrimental effect of active TB.

Based on our effect size analysis, we saw a meaningful improvement in HRQOL throughout treatment of active TB, and particularly during the initial, intensive phase.

Health care providers encounter detrimental effects of TB on HRQOL particularly in their patients with active TB disease, but to a lesser degree among those treated for LTBI. Indeed, one study documented poorer HRQOL among subjects treated for latent TB, compared to healthy controls [4]. These decrements are also highly relevant to decision makers, in approaching tradeoffs between providing preventive treatment for a large number of persons with LTBI, versus the smaller number who may ultimately develop active TB [28, 30].

It is worth noting that available studies primarily reflect the experiences of young and middle-aged, predominantly male adults—corresponding to the profile of reported TB cases in most countries [11]. Other socio-demographic characteristics of subjects varied widely, although data were sparse. Over 75 % of subjects with reported immigration status were immigrants to low TB incidence countries. Immigrants also face a unique set of challenges—adapting to new cultural norms and languages, obtaining and sustaining paid work, and accessing health care (for TB diagnosis/treatment and otherwise). These may all independently and synergistically affect HRQOL. Our results may therefore be particularly relevant to low-incidence countries, as immigrants represent the majority of persons screened, diagnosed, and treated for active TB disease and LTBI in such settings.

With a comprehensive search strategy yielding 76 articles, this systematic review builds on a review published in 2009 and is the first to provide pooled estimates from formal meta-analyses [22]. In this earlier review, subjects with active TB were also shown to have substantial deficits in HRQOL, compared to subjects treated for LTBI. Mental well-being was more severely disrupted than physical health among patients in both treatment groups. Our results are also similar with respect to improvement during treatment of active TB [22].

While 28 unique cohorts of patients with TB were included in our systematic review, 3 potentially relevant articles were excluded because they were published in Russian. Hence, our estimates of HRQOL may be affected by some selection bias. However, the main findings of our review were consistent across a variety of settings.

Some information could be gleaned from the English abstracts of two of these articles. Sukhov and Sukhov found that men with chronic pulmonary TB rated their HRQOL worse than men with their first case of pulmonary TB [44]. Shalaeva et al. [42] reported improvements in all sub-scales of the SF-36 among 59 adults who received surgical treatment and chemotherapy for pulmonary TB. This latter finding in particular supports findings of improved HRQOL scores throughout active TB treatment among the studies included in this review.

Only 2 studies retrieved from the search included patients who were co-infected with TB and HIV. Additionally, only 1 study included in this review evaluated subjects with multi-drug resistant TB (MDR-TB), and no studies evaluated children less than 11 years of age. Hence, our systematic review could not adequately address HRQOL among these groups, whose experience may differ substantially from that of other TB patients.

This systematic review synthesized the information from studies of *quantitative* evaluations of HRQOL; we did not review the body of research using qualitative methods to address HRQOL as it was beyond the scope of our study objectives. However, qualitative studies can offer health care providers valuable insight regarding patients’ experiences and needs in specific settings and should be used to supplement the quantitative information provided in this review.

The conclusions drawn from a systematic review depend on the quality of the individual studies included. One major limitation of the studies is that very few adequately measured and described key social and behavioral determinants of TB [11]. For this reason, our meta-analysis was limited to crude pooled estimates of HRQOL. We did not have sufficient information to permit meta-regression, which could help account for important confounders (e.g., foreign birth, substance use, co-morbidities) when comparing persons with active TB to those treated for LTBI and healthy control subjects.

Most studies were cross-sectional, and none reported measurements from a randomized clinical trial. Longitudinal observational studies can provide valuable insight into changes in HRQOL as patients undergo different phases of treatment, particularly for active TB. A longitudinal design, where subjects with active and LTBI are compared to suitable controls, will be particularly useful in this respect.

Inaccurate measures of HRQOL were possible as only 3 of the 28 cohorts included a process for checking questionnaire comprehension, and only one indicated the number of subjects excluded accordingly. Persons so excluded may have more limited educational attainment or language skills, which may make study samples less representative of the TB patient population, and perhaps lead to overestimation of HRQOL because of this exclusion. Additionally, one study contributing data to meta-analyses was not yet peer-reviewed [4]. Sensitivity analyses with removal of these data were performed accordingly, with generally similar findings.

Refusals ranged from 0 to 37 % in the 12 studies that provided this information. It is possible that subjects who refused to participate had more severe TB disease and/or a higher prevalence of risk behaviors, though this could not be assessed directly. These refusals could also make study samples less representative and potentially bias our results. Similarly, certain eligibility criteria might limit the representativeness of some studies.

Finally, most included studies appeared to be of moderate quality, with the most frequent quality score being 8 out of a possible 16 points. Although this rating system was not formally validated, it may highlight gaps in reporting parameters that are relevant to the assessment of HRQOL in the TB patient population.

## Conclusions

In a variety of studies, subjects with active TB consistently reported poorer HRQOL than persons treated for LTBI and untreated controls. This is important for understanding the non-fatal outcomes of active TB and the potential benefits of preventive interventions.

Future research on HRQOL in the TB context should better address social and behavioral health determinants. Further information is also needed for some of the most vulnerable persons with TB, such as those with TB/HIV co-infection, MDR-TB, and younger children.

In the TB context, meaningful cross-sectional differences and longitudinal changes have not been defined for many of the measurement tools we reviewed. A longitudinal study now underway compares HRQOL and health utilities over a 12-month follow-up period, among persons treated for active TB, LTBI, and untreated, healthy controls of similar background. This research will help address this gap in low-incidence settings, allow for better assessment of the benefits and limitations of TB control interventions, and may assist health care providers to better target physical and psychosocial support.

## Appendix 1

We used the following search strategy to retrieve relevant articles from eight databases.

((“Mycobacterium tuberculosis”[MeSH]) OR (“tuberculosis”[MeSH]) OR (“TB”[title/abstract])) AND ((“quality of life”[MeSH]) OR (“QoL”[title/abstract]) OR (“quality adjusted life years”[MeSH]) OR (“QALY”[title/abstract]) OR (“health utility*”[title/abstract]) OR (“health status”[MeSH]) OR (“life quality*”[title/abstract]) OR (“well-being”[title/abstract]) OR (“disability adjusted life year*”[title/abstract]) OR (“DALY*”[title/abstract]) OR (“treatment outcome”[MeSH]) OR (“outcome*”[title/abstract]) OR (“outcome assessment (health care)”[MeSH]) OR (“cost and cost analysis”[MeSH]) OR (“cost of illness”[MeSH]) OR (“disability*”[title/abstract]) OR (“health-related quality of life”[title/abstract]) OR (“HRQOL”[title/abstract]) OR (“ql”[title/abstract])).

## Appendix 2

The following information supplements the results published in the main manuscript relating to studies evaluating subjects with active TB. Refer to Table 6 in Appendix.

Active TB subjects consistently reported Short Form 36 (SF-36) Physical Component Scores (PCS) and Mental Component Scores (MCS) lower than concurrently evaluated patients treated for latent TB infection (LTBI) or healthy controls [3, 4, 17, 21, 29, 33, 35, 51, 52]. In the longitudinal studies, active TB patients rated their HRQOL better throughout their treatment, except for one study [3]. The previously treated subjects evaluated by Dion et al. [17] reported better or comparable physical and mental well-being than their active TB counterparts, but worse physical and mental well-being compared to the concurrently evaluated subjects treated for LTBI. Babikako et al. assessed HRQOL among smear-positive, pulmonary TB patients and TB/HIV co-infected individuals; as expected, the latter patient group reported lower PCS and MCS compared to the former except at the last evaluation. An additional study (Muniyandi et al. [35]) evaluated HRQOL of 436 subjects in India between 2003 and 2004 who were new cases of active TB, completed treatment, and were disease- and infection-free for 1 year since the completion of their active TB treatment regimen. Mean PCS and MCS scores demonstrated improved physical and mental well-being after 1 year of completing treatment compared to all active TB scores shown in Table 6 in Appendix [35].

Two studies used the GHQ-12 to assess HRQOL among subjects with active TB [1, 2]. Aghanwa et al. [1] examined active TB patients, long-stay hospitalized orthopedic patients, and healthy hospital workers in Nigeria; HRQOL improved across these three groups, respectively. Aydin et al. [2] assessed HRQOL in a Turkish population among incident cases of active TB, patients with active TB who have defaulted from previous treatment, and subjects with multi-drug resistant (MDR) TB. Incident active TB subjects rated their HRQOL better than defaulted or MDR-TB cases. However, a group of concurrently measured patients with Chronic Obstructive Pulmonary Disease (COPD) rated their HRQOL even worse than the patients with MDR-TB. Mean scores of each of the three TB patient groups rated their HRQOL better than the active TB subjects in the study by Aghanwa et al., which may be explained by the difference in socioeconomic status between Turkey and Nigeria.

Two studies used the Beck Depression Inventory (BDI) at assess HRQOL among subjects treated for active TB—Westaway et al. evaluated patients hospitalized in South Africa and Unalan et al. [46, 47, 53] assessed Turkish patients treated for active TB, patients treated for LTBI, and a healthy control group. All subjects treated for TB reported worse HRQOL compared to the healthy controls and met the cut-off for depression, but the South African patients rated their HRQOL better than both treated groups of Turkish patients.

Two studies used the EQ-5D instrument to measure health utilities among subjects treated for active TB [17, 29]. The mean score reported by subjects evaluated by Dion et al. [17] indicated better HRQOL compared to those evaluated by Kruijshaar et al. In the former study, those treated for LTBI and previously treated subjects reported the same health utilities. These results indicate that in a low-prevalence setting, health utilities among those treated for active TB may improve to the level of a patient treated with preventative treatment after 6 months of completing therapy for active TB disease. Subjects treated for active TB in the study by Kruijshaar et al. indicated similar improvements in health utilities throughout treatment as those reported with the SF-36.

When measuring HRQOL among active TB subjects in Pakistan with the Hospital Anxiety and Depression Scale (HAD), Husain et al. [26] found that 47 % of their subjects met the anxiety criteria score, and 46 % of their subjects met the depression criteria score.

Two studies calculated the SF-6D metric from data collected with the SF-36 [4, 21]. Mean SF-6D scores among subjects treated for active TB were about 0.10 points lower than those treated for LTBI within 2 weeks of TB diagnosis. Health utilities reported by healthy controls were identical to those reported by subjects treated for LTBI [4]. Although studied after one to 2 months of treatment, patients of the Guo et al. study reported similar SF-6D mean scores to those patients evaluated by Bauer et al.

Subjects treated for active TB in the study by Dion et al. reported health utilities measured with the Standard Gamble that indicated better HRQOL than those subjects treated for active TB studied by Bauer et al. [4, 16]. Similar health utilities were reported by concurrently studied subjects treated for LTBI. Perfect health was reported by the healthy controls in the Bauer et al. study as well as the subjects previously treated for active TB in the Dion et al. study. Although the sample size was limited in the Dion et al. study, these results may indicate a return to excellent health utility within 6 months of completing active TB treatment in a low-prevalence setting.

Unalan et al. and Kruijshaar et al. [29, 45, 46] used the STAI-6 to evaluate HRQOL among patients with active TB. Unalan et al. [45, 46] found that subjects treated for active TB reported the same HRQOL as the concurrently evaluated subjects treated for LTBI. Similar to patterns found with the SF-36 and the EQ-5D, subjects treated for active TB in the study by Kruijshaar et al. [29] reported an improvement in HRQOL with STAI-6 throughout treatment.

Two studies examined HRQOL among Canadian patients with active TB using the VAS—Guo et al. [16, 21] used a 10-cm ‘feeling thermometer’ with 0 cm = ‘death’ and 10 cm = ‘perfect health,’ and Dion et al. used a 100-cm scale with 0 cm = ‘death’ and 100 cm = ‘perfect health’. In both studies, participants were asked to mark on the scale how they rated their own health state at the time of the interview. The mean scores of the active TB patients evaluated by Dion et al. were 12 points greater than the mean score reported by the subjects studied by Guo et al. [16, 21]. Side effects from active TB treatment would not have occurred within 2 weeks of TB diagnosis, which likely explains the differences in the mean scores between these 2 studies. Mean scores reported by those subjects treated for LTBI were similar in the 2 studies and also similar to the mean score reported by those subjects previously treated for active TB in the study by Dion et al. [16, 21]. This supports the notions that (1) even after 2 months of LTBI treatment, subjects do not see a deficit in HRQOL, (2) subjects treated for active TB report far worse HRQOL than those treated for LTBI (as was seen in multiple studies that assessed HRQOL using the SF-36), and (3) subjects who complete active TB treatment may return to a level of HRQOL similar to individuals treated for LTBI.

The Brief Disability Questionnaire (BDQ) was used by Aydin et al. [2] who found a downward gradation in HRQOL from new cases of active TB, to persons with active TB who had defaulted from treatment, to patients with MDR-TB. The group of subjects with COPD, however, rated their HRQOL much worse than even the group of patients with MDR-TB. This trend was similar to that found when using the GHQ-12 as described above.

The Center for Epidemiologic Studies Depression Scale (CES-D) used by Kruijshaar et al. [29] indicated that the HRQOL improved from TB diagnosis to 2 months of completed TB treatment among active TB subjects. These results were similar to those found with the SF-36, EQ-5D, and STAI-6 [29].

Dhingra et al. [14, 15] used the DR-12 to assess HRQOL among active TB subjects at the beginning of TB treatment, at the completion of the initial phase of TB treatment, and at the completion of the TB treatment regimen. Subjects reported improved HRQOL throughout the course of TB treatment.

The multi-attribute health utility indices (HUI2 and HUI3, score ranges from 0 to 1) were used by the Guo et al. [21] to evaluate both active TB patients and patients treated for LTBI. Health utilities for the active TB patients were 0.09 points lower with the HUI3 than with the HUI2. On average, the subjects treated for LTBI reported health utilities 0.08 points higher than active TB patients on the HUI2 and 0.14 higher on the HUI3 [21].

Deribew et al. [12] used the Kessler 10 to compare HRQOL among subjects with TB/HIV to HIV-positive individuals without active TB, referred to as ‘HIV-only’ subjects. All subjects were hospitalized at HRQOL evaluation. All TB/HIV subjects were within the initial phase of TB treatment, and 75 % of these subjects were also taking anti-retroviral therapy (ART). All HIV-only patients were taking ART. Among the patients classified by the authors to be in good physical health, a greater proportion of the TB/HIV subjects were classified with depression than the HIV-only patients [12]. These results are similar to those reported by TB/HIV co-infected patients evaluated by Babikako et al. with the SF-36 [29].

Two different studies conducted in China examined HRQOL among subjects with active TB and those treated for LTBI [20, 59]. Fu et al. [20] found that active TB subjects rated their HRQOL worse than the patients treated for LTBI when using the Life Satisfaction Index Z, the SAS, or the SDS. Although Yang et al. [59] found no difference in mean SSRS scores reported by subjects treated for active TB and those treated for LTBI, active TB subjects rated their HRQOL more than 10 points worse than concurrently assessed subjects treated for LTBI when using the SCL-90.

Rajeswari et al. [40] evaluated active TB patients in India at 3 different time points with a modified version of the SF-36: at TB diagnosis, after completing 2 months of TB treatment, and after treatment completion. Table 6 in Appendix shows a wide range of the proportion of subjects reporting good HRQOL on physical, mental, and social domains across the 3 evaluations [40].

Maguire et al. [31] used a modified version of the SGRQ to evaluate subjects treated for active TB at diagnosis, and after completing 2 and then 6 months of treatment. Similar to other studies reported above, HRQOL reported by these patients improved throughout the course of treatment [31].

Aghanwa et al. [1] found a greater proportion (30 %) of subjects with active TB were classified as having a psychiatric disorder when using the Present State Examination (PSE) compared to subjects treated for LTBI (10 %) and long-term hospitalized orthopedic patients (5 %).

After 6–8 months of TB treatment, subjects treated for LTBI reported better HRQOL than those subjects treated for active TB, as measured by Pasipanodya et al. [37] using the SGRQ.

Fourteen patients with laryngeal active TB were evaluated by Yelken et al. [60] with the VHI-10 questionnaire at the start of treatment and during a follow-up visit, which occurred within 6 weeks to 9 months of completing treatment. HRQOL improved from the start of treatment to the completion of treatment in this sample [60].

Dhuria et al. [15] used the World Health Organization’s Quality of Life Questionnaire—BREF (WHOQOL-BREF) to assess HRQOL among subjects with active TB in India at the start of TB treatment, after completing 3 months of TB treatment, and after completing the entire regimen. Similar to numerous studies described above, mean HRQOL scores improved from the initial evaluation through the 2 follow-up assessments [15]. However, the mean HRQOL scores of the active TB patients never reached the mean scores of LTBI-treated subjects assessed at the start of their TB treatment.

Contrary to their results found with the Kessler 10, Deribew et al. [12] found that the range of WHOQOL-HIV scores for the TB/HIV subjects indicated slightly *better* HRQOL than the HIV-only group. However, after controlling for a number of confounding variables such as age, sex, occupation, CD4 lymphocyte count in a multivariable model, TB/HIV subjects had lower mean scores in all domains of the WHOQOL-HIV [12]. Significantly greater proportion of TB/HIV subjects had lower CD4 lymphocyte counts compared to HIV-only subjects (57.5 % compared to 27.3 %, respectively), which may contribute to the lower HRQOL among the former group [12].

## Appendix 3

The following information supplements the results presented in Table 4.

Three studies evaluated HRQOL among active TB patients at three time points roughly at the beginning of TB treatment, at the end of the initial intensive phase of treatment, and well into the continuation phase of treatment or at the end of treatment [13–15, 32]. In all three studies, the greatest improvement in HRQOL occurred during the first 2–3 months of treatment. Based on the previously published minimum important difference of 4 points for the SGRQ, 94 % of subjects reported by Maguire et al. [27, 32] had a meaningful improvement between diagnosis and 2 months of treatment; between 2 and 6 months of treatment, 80 % of these subjects reported meaningful improvement.

Kruijshaar et al. [29] used 3 instruments to evaluate HRQOL among patients treated for active TB at diagnosis and after 2 months of treatment. Although there was some improvement in HRQOL as indicated by the effect sizes for the Short Form 36 (SF-36) Physical Component Score (PCS) and the State-Trait Anxiety Inventory Short Form (STAI-6), these effects sizes did not indicate meaningful change in HRQOL. However, meaningful improvements in HRQOL were suggested by the effect sizes of the SF-36 Mental Component Score (MCS) and the Center for Epidemiologic Studies Depression Scale (CES-D) [29].

Marra et al. [33] measured HRQOL with the SF-36 and the Beck Depression Inventory (BDI) among patients treated for active TB at their diagnosis and after completing 6 months treatment. There was no meaningful change indicated by the effects size for all three instruments. Our calculations match the conclusion made by Marra et al. [33, 42], who used a standard of the difference in the two mean scores of at least 5 points to identify meaningful changes in mean HRQOL scores.

Marra et al. [33] also provided longitudinal measures of the SF-36 and the BDI among subjects treated for LTBI. None of the effect sizes indicated meaningful changes in HRQOL. SF-36 results were confirmed by the standard of using a 5-point cut-off of mean scores to determine meaningful changes in HRQOL [42].

## Appendix 4

The following information summarizes results of 4 studies assessing HRQOL among subjects with post-TB sequelae, i.e, no longer active TB disease [9, 10, 19, 37, 39, 56, 57]. These patients developed chronic alveolar hypoventilation and were subsequently placed on home mechanical ventilation (HMV) (Table 7 in Appendix). Five HRQOL instruments were used among these four studies.

The GHQ-12 was used in 2 studies; in both studies, post-TB sequelae patients rated their HRQOL better than the comparison groups of patients on HMV due to other diseases [9, 10, 19, 37, 39]. HRQOL among these post-TB sequelae patients was, however, rated lower than the active TB subjects and comparison groups assessed by Aydin et al. and Aghanwa et al. [1, 2] with the same questionnaire. Patients with post-TB sequelae are much older than the active TB patients evaluated by Aydin et al. and Aghanwa et al., and thus, their HRQOL may be affected by age and other factors.

Pehrsson et al. [39] found that the group of post-TB sequelae patients (compared to the group of subjects also on HMV due to other diseases) reported better HRQOL according to their mean HAD score for anxiety, but worse HRQOL according to their mean HAD scores for depression.

Using the Mood Adjective Checklist Short Form (MACL), Dellborg et al. [9, 10, 19, 36] observed noticeably worse HRQOL among the patients on HMV due to post-TB sequelae compared to the group of patients on HMV due to other diseases. The range of mean MACL domain scores found by Pehrsson et al. [39] did not, however, indicate a difference in HRQOL between these 2 groups of patients.

Dellborg et al. and Pehrsson et al. [9, 10, 19, 37, 39] also used the Sickness Impact Profile (SIP) to evaluate HRQOL of patients on HMV. Dellborg et al. found worse mean SIP scores among the group of subjects on HMV due to post-TB sequelae compared to patients on HMV due to other diseases, with the former group exceeding the score cut-off of severe dysfunction [9, 10, 19, 37]. On the contrary, the mean SIP scores in the study by Pehrsson et al. [39] indicated that the post-TB sequelae patients rated their HRQOL better than the comparators.

Two studies used the Severe Respiratory Insufficiency Questionnaire (SRI) [31, 56, 57]. One study found worse HRQOL among subjects on HMV due to post-TB sequelae compared to subjects on HMV due to other diseases [56, 57]. The second study found that patients on HMV due to post-TB sequelae rated their HRQOL slightly better than patients on HMV for other illnesses [31]. However, there was only a 7-point difference between the highest mean score and lowest mean score of these patient groups across the 2 studies.

**Table 5:** Description of HRQOL and health utility instruments used by studies included in the reviewed literature

Instrument	Number of studies using	Description
SF-36 Health Survey (SF-36)	8	36 items measuring 8 health domains (physical functioning, role limitations due to physical health problems, bodily pain, general health, vitality, social functioning, role limitations due to emotional problems, mental health). Scores range from 0 to 100 with greater scores indicating better HRQOL
General Health Questionnaire 12 (GHQ 12)	4	Modified from the General Health Questionnaire 60 and contains 12 items, each ranked on a 4-point Likert scale ranging from 0 = ‘absent’ to 3 = ‘present.’ Higher total scores indicate worse HRQOL
Beck Depression Inventory (BDI)	2	21-item in multiple choice format measuring the presence and degree of depression among adolescents and adults. Numerical values of 0, 1, 2, or 3 are assigned to each statement to indicate degree of severity. Items are summed to produce an overall score—a cut-off score of ≥13 indicates depression, with greater scores indicating more severe depression
Euro-QoL (EQ 5D)	2	5 domains (mobility, self-care, usual activities, pain/discomfort and anxiety/depression). Each item is ranked on a 3-point scale ranging from ‘no problems’ to extreme problems’ with higher scores indicating better HRQOL
Hospital Anxiety and Depression Scale (HAD)	2	14 items comprising 2 dimensions—anxiety and depression. Scores for each item ranges from 0 to 3, with higher scores indicating worse HRQOL (i.e. more anxiety and depression)
Mood Adjective Check List Short Form (MACL)	2	38 items categorized into 3 dimensions (pleasantness, activation, calmness). Scores for each item range from 1 to 4, with higher scores indicating better HRQOL
Severe Respiratory Insufficiency Questionnaire (SRI)	2	49 items comprising 7 domains (respiratory complaints, physical functioning, attendant symptoms and sleep, social relationships, anxiety, psychological well-being, social functioning). Items are rated on a 5-point Likert scale from ‘strongly disagree’ to ‘strongly disagree’. Higher scores indicate better HRQOL
SF-6D utility score	2	11-item generic preference-based single index measure of health status. Scores range from 0 to 1.0, with higher scores indicating better HRQOL
Sickness Impact Profile (SIP)	2	136 items measuring 12 domains (sleep and rest, emotional behavior, body care and movement, home management, mobility, social interaction, ambulation, alertness, behavior, communication, work, recreation and pastimes, eating). Subjects endorse items that describe themselves. Scores are calculated as a percentage of total dysfunction; a cut-off score > 10 indicates severe dysfunction
Standard Gamble	2	Subject choose between certainty of remaining in a given health state and a hypothetical gamble, between the possible outcomes of perfect health and death. Probability of having perfect health in the gamble is lowered from 100% until the subject is indifferent between the choices. (Administration assisted with the use of a colored probability wheel.) The midpoint of the values between this probability of perfect health and the previous probability is the HRQOL score. Scores range from 0 to 1, with higher scores indicating better HRQOL
State-Trait Anxiety Inventory Short Form (STAI-6)	2	6 items are rated on a 4-point scale. (Created from the 40-item Spielberger State-Trait Anxiety Inventory.) Items are scaled using a 4-point Likert scale indicating levels of anxiety with 1 = ‘not at all’ to 4 = ‘very much.’ Total scores range from 20 to 80 with a cut-off total score > 44 demonstrating that the subject is highly anxious. (Higher scores indicate worse HRQOL)
Visual Analog Scale (VAS)	2	Two methods used: (1) 10-cm scale with 0 cm = ‘death’ and 10 cm = ‘perfect health’ and (2) 100-cm ‘feeling thermometer’ with 0 cm = ‘death’ and 100 cm = ‘perfect health’. Patients are asked to mark on these scales where they rate their own state of health. Higher scores indicate better HRQOL
Beck Depression Inventory (BDI Short Form)	1	13 items in multiple choice format measuring the presence and degree of depression among adolescents and adults. Numerical values of 0, 1, 2, or 3 are assigned to each statement to indicate degree of severity. Items are summed to produce an overall score with cut-offs of 0-3 = none or minimal depression, 4–7 = mild depression, 8–15 = moderate depression, and ≥16 = severe depression. (Higher scores indicate worse HRQOL)
Brief Disability Questionnaire (BDQ)	1	11 items scored on a scale from 0 = never to 2 = always or severe; higher scores indicate worse HRQOL
Center for Epidemiologic Studies Depression Scale (CES-D)	1	15 items each ranked on a 4-point Likert scale ranging from ‘not at all’ to ‘very much’. Total scores range from 0 to 60, with a score cut-off ≥16 suggesting a clinically significant level of depressive symptoms. (Higher scores indicate worse HRQOL)
DR-12	1	12 items comprising 2 domains—symptoms and socio-psychological & exercise adaptation. Each item is ranked on a scale from 1 to 3; higher scores indicate better HRQOL
Duke Health Profile (DUKE)	1	63 items comprising 4 dimensions (symptom status, physical function, social function, and emotional function). Scores are generated for each domain is scored on a scale of 0–1; higher scores indicate better HRQOL
Dysfunctional Analysis Questionnaire (DAQ)	1	50 items comprising 5 domains (social, vocational, personal, familial, cognitive). Each item rated on a 5-point Likert scale with 1 = better functioning than that before onset of illness and 5 = severe impairment compared to before illness onset; higher scores indicate worse HRQOL
Health Utilities Index 2 (HUI 2)	1	7 items (sensation, mobility, emotion, cognition, self-care, pain, fertility) each with 3 to 5 levels. Utility functions are assigned to each level of each item from which that total health utility is calculated that ranges from 0 (death) to 1 (perfect health)
Health Utilities Index 3 (HUI 3)	1	8 items (vision, hearing, speech, ambulation, dexterity, emotion, cognition, pain) each with 5 to 6 levels. Utility functions are assigned to each level of each item from which that total health utility is calculated that ranges from 0 (death) to 1 (perfect health).
Kessler 10	1	10 items each ranked on a 5-point Likert scale ranging from 1 = ‘never’ to 5 = ‘all of the time’. Higher scores indicate better HRQOL
Life Satisfaction Index Z	1	A short form version of the Life Satisfaction Index A containing 13 items. Subjects are asked to agree or disagree with the statement. Total scores range from 0 to 26, with higher scores indicating greater HRQOL
Modified version of SF-36	1	14 items among 3 domains (physical well-being, mental well-being, and social well-being). Responses are categorized into 1 of 3 categories: “worse,” “same as before,” and “better”. Total scores range from 0 to 100; higher scores indicate better HRQOL
Modified version of St. George’s Respiratory Questionnaire (SGRQ)	1	A version of the SGRQ suited to the study population, located in the Timika District, Papua Province, Indonesia. For more details refer to the SGRQ below
Present State Examination (PSE)	1	A combination of the 30-item General Health Questionnaire and a Self-rating Depression Scale. The 30-item General Health Questionnaire is a modified version of the General Health Questionnaire 60 and contains 30 items, each ranked on a 4-point Likert scale ranging from 0 = ‘absent’ to 3 = ‘present.’ Higher total scores indicate worse HRQOL. The Self-rating Depression Scale is designed to assess the level of depression among individuals diagnosed with a depressive disorder. There are 20 items comprising 4 domains (the pervasive effect, the physiological equivalents, other disturbances, and psychomotor activities). Each item is ranked on a scale of 1 = ‘a little of the time’ to 4 = ‘most of the time’. Higher scores indicate worse HRQOL with cut-off scores of 20-49 = normal range, 50–59 = mildly depressed, 60–69 = moderately depressed, ≥70 = severely depressed
Rosenberg Self-Esteem Scale (RSE)	1	10 items with each item ranked on a 4-point Likert scale from ‘strongly agree’ to ‘strongly disagree’; higher scores indicate better HRQOL
Self-Rating Anxiety Scale (SAS)	1	20 items each ranked on 4-point, Likert scale where 1 = ‘a little of the time’ and 4 = “most of the time. Total scores range from 20–80 with score classifications: 20–44 points = normal range anxiety; 45–59 points = mild to moderate anxiety levels; 60–74 points = marked to severe anxiety levels; 75–80 extreme anxiety levels. Higher scores indicate worse HRQOL
Sheehan Disability Scale (SDS)	1	20 items comprising 3 domains: work, family, and social lives. Each item rated by using a 10-point visual analog scale with 0 = ‘unimpaired’ to 10 = ‘highly impaired.’ Total scores range from 0 to 30; higher scores indicate worse HRQOL. Scores ≥5 in any of the 3 domains indicates significant impairment
Social Support Rating Scale (SSRS)	1	10 items to assess the perceived helpfulness of different types of individuals to the subject. Each item is assessed using a 3-point, Likert scale ranging from 1 = ‘not at all helpful’ to 3 = ‘a great deal helpful’. Higher scores indicate more social support
St. George Respiratory Questionnaire Short Form (SGRQ)	1	A disease-specific instrument designed to assess patients with mild to severe airway disease. 50 items comprise 3 domains (symptoms, activity, and impacts). Scores are scaled from 0 to 100, with better scores indicating worse HRQOL
Symptoms Check List (SCL-90)	1	90 items comprising 9 domains (somatization, obsessive-compulsive, interpersonal sensitivity, depression, anxiety, anger-hostility, phobic anxiety, paranoid ideation, psychoticism). Three global indices (Global Severity Index, Positive Symptom Total, and Positive Symptom Distress Index) can also be calculated. Each item is ranked on a 5-point Likert scale, ranging from 0 = `not at all’ to 4 = `extremely’; higher scores indicating worse HRQOL
Voice Handicap Index-10 (VHI-10)	1	Assessment specific to voice handicap. 30 items comprising 3 domains (functional, physical, and emotional aspects of voice disorders). Each item is ranked on a 5-point Likert scale with 0 = never to 4 = always; higher scores indicate worse HRQOL
World Health Organization’s Quality of Life—BREF (WHOQOL-BREF)	1	Based on the WHOQOL-100. 26 items across 5 domains (physical health, psychological health, social relationships, environment) are ranked on a 5-point Likert scale ranging from 1 = ‘not at all’ to 5 = ‘an extreme amount’. Higher scores indicate better HRQOL
World Health Organization’s Quality of Life—HIV (WHOQOL-HIV)	1	Based on the WHOQOL-100 questionnaire, to be used for patients with HIV/AIDS. 115 items (comprising 30 facets) are each ranked on a 5-point Likert scale ranging from 1 = ‘not at all’ to 5 = ‘an extreme amount’. Higher scores indicate better HRQOL

**Table 6:** Mean HRQOL and health utility scores reported by studies evaluating subjects treated for active TB and their comparison patient groups

Instrument	Study (reference number)	Year of data collection	Country	Time of assessment and associated measures	Patient groups			
					*Active TB*	*LTBI*	*Healthy Controls*	*Other*
SF-36								
	Babikako et al. [3]	2007–2008	Uganda	Within 2 weeks of TB diagnosis, N	23	0	0	20 TB/HIV co-infected patients
				Mean PCS (95 % CI)	61 (52,70)	–	–	55 (NA)
				Mean MCS (95 % CI)	61 (53,69)	–	–	59 (NA)
				One to two months of TB treatment, N	19	0	0	20 TB/HIV co-infected patients
				Mean PCS (95 % CI)	70 (61, 79)	–	–	64 (NA)
				Mean MCS (95 % CI)	72 (65,79)	–	–	67 (NA)
				6–8 months of TB treatment, N	23	0	0	20 TB/HIV co-infected patients
				Mean PCS (95 % CI)	65 (55, 75)	–	–	77 (NA)
				Mean MCS (95 % CI)	68 (59, 77)	–	–	80 (NA)
	Bauer et al. [4]	2008–2011	Canada	Within 2 weeks of TB diagnosis, N	61	119	77	0
				Mean PCS (95 % CI)	49 (47, 51)	56 (55, 57)	57 (56, 58)	–
				Mean MCS (95 % CI)	44 (41, 47)	50 (49, 51)	50 (48, 52)	–
	Dion et al. [17]	1999–2000	Canada	Within 2 weeks of TB diagnosis, N	17	25	0	8 patients 6 months post-TB treatment completion
				Mean PCS (95 % CI)	53 (49, 57)	58 (56, 60)	–	56 (40, 72)
				Mean MCS (95 % CI)	49 (47, 57)	57 (53, 62)	–	49 (23, 74)
	Guo et al. [21]	2008^a^	Canada	One to two months of TB treatment, N	84	78	0	0
				Mean PCS (95 % CI)	45 (42, 48)	55 (53, 56)	–	–
				Mean MCS (95 % CI)	40 (37, 43)	50 (49, 52)	–	–
	Kruijshaar et al. [29]	2008	England	Within 2 weeks of TB diagnosis, N	42	0	0	0
				Mean PCS (95 % CI)	36 (32, 40)	–	–	–
				Mean MCS (95 % CI)	42 (38, 45)	–	–	–
				One to two months of TB treatment, N	31	0	0	0
				Mean PCS (95 % CI)	39 (35, 43)	–	–	–
				Mean MCS (95 % CI)	50 (46, 53)	–	–	–
	Marra et al. [33]	2005–2006	Canada	Within 2 weeks of TB diagnosis, N	104	102	0	0
				Mean PCS (95 % CI)	48 (45, 50)	55 (52, 57)	–	–
				Mean MCS (95 % CI)	43 (40, 45)	52 (49, 54)	–	–
				Six to eight months of TB treatment, N	70	75	0	0
				Mean PCS (95 % CI)	49 (46, 51)	54 (53, 56)	–	–
				Mean MCS (95 % CI)	46 (45, 49)	50 (48, 52)	–	–
	Wang et al. [51, 52]	1996	China	One to two months of TB treatment, N	228	0	228	0
				Mean PCS (95 % CI)	65 (61, 68)	–	84 (81, 87)	–
				Mean MCS (95 % CI)	61 (58, 64)	–	74 (72, 76)	–
GHQ-12								
	Aghanwa et al. [1]	1995–1996	Nigeria	Unspecified time during TB treatment, N	53	0	20	20 long-stay orthopedic patients
				Mean total score (95 % CI)	3.5 (2.7, 4.2)	–	1.8 (1.3, 2.3)	1.9 (0.9, 2.9)
	Aydin et al. [2]	1999	Turkey	Unspecified time during TB treatment, hospitalized (new/default/MDR), N	42/38/39	0	0	38 COPD patients
				Mean total score new/default/MDR (95 % CI)^b^	0.94/2.0/2.0 (0.7, 1.2/1.4,2.6/1.5,2.5)	–	–	3.4 (2.6, 4.2)
BDI								
	Westaway et al. [53]	1992^a^	South Africa	Unspecified time during TB treatment, hospitalized, N	100	0	0	0
				Mean total score (95 % CI)	13.6 (11.9, 15.2)	–	–	–
	Unalan et al. [45, 46]	2003–2004	Turkey	Unspecified time during TB treatment, N	196	108	196	0
				Mean total score (95 % CI)	17.5 (15.9, 19.1)	17.4 (15.1, 19.7)	9.1 (8.3, 9.8)	–
EQ-5D								
	Dion et al. [17]	1999–2000	Canada	Within 2 weeks of TB diagnosis, N	17	25	0	8 subjects 6 months post-TB treatment completion
				Mean total score (95 % CI)	79 (68, 90)	88 (81, 95)	–	88 (62, 100)
	Kruijshaar et al. [29]	2008	England	Within 2 weeks of TB diagnosis, N	61	0	0	0
				Mean total score (95 % CI)	67 (66, 68)	–	–	–
				One to two months of TB treatment, N	55	0	0	0
				Mean total score (95 % CI)	81 (NA)	–	–	–
HAD								
	Husain et al. [26]	2008^a^	Pakistan	Unspecified time during TB treatment, N	108	0	0	0
				*N* (%) classified with anxiety (HAD score ≥11)	51 (47)	–	–	–
				*n* (%) classified with depression (HAD score > 11)	50 (46)	–	–	–
SF-6D utility score								
	Bauer et al. [4]	2008–2011	Canada	Within 2 weeks of TB diagnosis, N	61	119	77	0
				Mean total score (95 % CI)	0.71 (0.67, 0.75)	0.81 (0.79, 0.83)	0.81 (0.79, 0.83)	–
	Guo et al. [21]	2008^a^	Canada	One to two months of TB treatment, N	84	78	0	0
				Mean total score (95 % CI)	0.68 (0.65, 0.72)	0.82 (0.80, 0.85)	–	–
Standard gamble								
	Bauer et al. [4]	2008–2011	Canada	Within 2 weeks of TB diagnosis, N	61	119	77	0
				Mean total score (95 % CI)	0.7 (0.6, 0.8)	0.9 (0.8, 0.9)	1.0 (0.98, 1.0)	–
	Dion et al. [16]	1999–2000	Canada	Within 2 weeks of TB diagnosis, N	17	25	0	8 subjects 6 months post-TB treatment completion
				Mean total score (95 % CI)	0.9 (0.8, 0.9)	0.9 (0.80, 1.0)	–	1.0 (0.90, 1.0)
STAI-6								
	Kruijshaar et al. [29]	2008	England	At TB diagnosis, N	61	0	0	0
				Mean total score (95 % CI)	57 (44, 53)	–	–	–
				One to two months of TB treatment. N	55	0	0	0
				Mean total score (95 % CI)	47 (40, 48)	–	–	–
	Unalan et al. [45, 46]	2003–2004	Turkey	Unspecified time during TB treatment, N	196	108	196	0
				Mean total score (95 % CI)	50 (41.4, 57.6)	50 (43.0, 57.3)	49.7 (42.9, 56.5)	–
VAS								
	Dion et al. [16]	1999–2000	Canada	Within 2 weeks of TB diagnosis, N	17	25	0	8 subjects 6 months post-TB treatment completion
				Mean total score (95 % CI)	78 (49, 100)	89 (71, 100)	–	88 (68, 100)
	Guo et al. [21]	2008^a^	Canada	One to two months of TB treatment, N	84	78	0	0
				Mean total score (95 % CI)	66 (61, 71)	87 (84, 90)	–	–
BDI short form								
	Westaway et al. [53]	1992^a^	South Africa	Unspecified time during TB treatment, hospitalized, N	100	0	0	0
				Mean total score (95 % CI)	8.0 (7.0, 9.1)	–	–	–
BDQ								
	Aydin et al. [2]	1999	Turkey	Unspecified time during TB treatment, hospitalized (new/default/MDR), N	42/38/39	0	0	38 COPD patients
				Mean total score new/default/MDR (95 % CI)^b^	2.8/2.9/5.7 (1.5, 4.1/1.8, 4.0/4.5, 6.9)	–	–	9.0 (7.6, 10.4)
CES-D								
	Kruijshaar et al. [29]	2008	England	At TB diagnosis, N	61	0	0	0
				Mean total score (95 % CI)	22 (18, 25)	–	–	–
				One to two months of TB treatment, N	61	0	0	0
				Mean total score (95 % CI)	13 (8, 18)	–	–	–
DR-12								
	Dhingra et al. [13, 14]	2002	India	Beginning of TB treatment, N	43	0	0	0
				Mean total score (95 % CI)	25.6 (24.3, 26.9)	–	–	–
				Completion of initial phase TB treatment, N	43	0	0	0
				Mean total score (95 % CI)	33.1 (32.3, 33.9)	–	–	–
				At TB treatment completion, N	43	0	0	0
				Mean total score (95 % CI)	34.6 (34.1, 35.0)	–	–	–
DUKE								
	Vinaccia et al. [49]	2007^a^	Colombia	Unspecified time during TB treatment, N	60	0	0	0
				Mean total score (95 % CI)	14.3 (13.4, 15.2)	–	–	–
DAQ								
	Bhatia et al. [6]	2001^a^	India	Unspecified time during TB treatment, N	50	0	0	0
				Range domain mean scores	70.8–86.4	–	–	–
HUI 2								
	Guo et al. [21]	2008^a^	Canada	One to two months of TB treatment, N	84	78	0	0
				Mean total score (95 % CI)	0.85 (0.80, 0.89)	0.93 (0.90, 0.95)	–	–
HUI 3								
	Guo et al. [21]	2008^a^	Canada	One to two months of TB treatment, N	84	78	0	0
				Mean total score (95 % CI)	0.76 (0.70, 0.82)	0.90 (0.86, 0.94)	–	–
Kessler 10								
	Deribew et al. [12]	2009	Ethiopia	Within the initial phase TB treatment, hospitalized & HIV co-infected with 75 % subjects on ART, N	467	0	0	155 HIV-only patients on ART
				*n* (%) depressed among those in good physical health	7 (37)	–	–	59 (27)
				*n* (%) depressed among those in poor physical health	12 (63)	–	–	159 (73)
Life Satisfaction Index Z								
	Fu et al. [20]	2001–2004	China	Unspecified time during TB treatment, N	510	0	100	0
				Mean total score (95 % CI)	7.2 (6.9, 7.5)	–	9.1 (8.5, 9.7)	–
Modified SF-36								
	Rajeswari et al. [40]	2001	India	At TB diagnosis. N	610	0	0	0
				Range of n (%) reporting good HRQOL (physical/mental/social domains)	34 (5.5)–484 (79)/101 (17)–373 (61)/347 (57)–562 (92)	–	–	–
				After initial intensive phase TB treatment. N	610	0	0	0
				Range of n (%) reporting good HRQOL (physical/mental/social domains)	156 (26)–209 (34)/160 (26)–566 (93)/351 (56)–562 (92)	–	–	–
				At TB treatment completion. N	610	0	0	0
				Range of *n* (%) reporting good HRQOL (physical/mental/social domains)	85 (14)–483 (79)/325 (53)–584 (96)/365 (60)–562 (92)	–	–	–
Modified SGRQ								
	Maguire et al. [32]	2003–2004	Indonesia	At TB diagnosis, N	115	0	0	0
				Mean total score (95 % CI)	44.6 (40.2, 48.9)	–	–	–
				One to two months of TB treatment, N	65	0	0	0
				Mean total score (95 % CI)	19 (17.0, 21.0)	–	–	–
				Six to eight months of TB treatment, N	66	0	0	0
				Mean total score (95 % CI)	7 (5.0, 9.0)	–	–	–
PSE								
	Aghanwa et al. [1]	1995–1996	Nigeria	Unspecified time during TB treatment, N	53	0	20	20 long-stay orthopedic patients
				*n* (%) of patients with a psychiatric disorder	16 (30)	–	2 (10)	1 (5)
RSE								
	Westaway et al. [53]	1992^a^	South Africa	Unspecified time during TB treatment, hospitalized, N	100	0	0	0
				Mean total score (95 % CI)	28.0 (26.8, 29.2)	–	–	–
SAS								
	Fu et al. [20]	2001–2004	China	Unspecified time during TB treatment, N	510	0	100	0
				Mean total score (95 % CI)	39.8 (39.1, 40.5)	–	36.4 (35.1, 37.7)	–
SDS								
	Fu et al. [20]	2001–2004	China	Unspecified time during TB treatment, N	510	0	100	0
				Mean total score (95 % CI)	43.7 (43.1, 44.3)	–	39.9 (38.6, 41.2)	–
SSRS								
	Yang et al. [59]	2003	China	Unspecified time during TB treatment, hospitalized, N	132	0	71	0
				Mean total score (95 % CI)	36.5 (NA)	–	36.5	–
SGRQ								
	Pasipanodya et al. [38]	2005–2006	USA	Six to eight months of TB treatment, N	105	199	0	0
				Mean total score (95 % CI)	24 (19.6, 28.4)	10 (8.0, 12.0)	–	–
SCL-90								
	Yang et al. [59]	2003	China	Unspecified time during TB treatment, hospitalized, N	132	0	71	0
				Mean total score (95 % CI)	36.1 (NA)	–	24.9	–
VHI-10								
	Yelken et al. [60]	2008^a^	Turkey	At start of TB treatment, N	14	0	0	0
				Median score (range)	24 (22–24)	–	–	–
				Six to eight months of TB treatment, N	14	0	0	0
				Median score (range)	12 (9, 15)	–	–	–
WHOQOL-BREF								
	Dhuria et al. [15	2004–2005	India	At start of TB treatment, N	90	0	90	0
				Mean total score (95 % CI)	11.8 (11.5, 12.0)	–	14.2 (14.0, 14.4)	–
				Three months of TB treatment, N	90	0	Not available	0
				Mean total score (95 % CI)	13.2 (13.0, 13.5)	–	–	–
				At treatment completion, N	90	0	Not available	0
				Mean total score (95 % CI)	13.9 (13.7, 14.2)	–	–	–
WHOQOL-HIV								
	Deribew et al. [12]	2009	Ethiopia	Within the initial phase TB treatment, hospitalized & HIV co-infected, N with 75 % subjects on ART	467	0	0	155 HIV-only patients on ART
				Range mean domain scores	12.4–17.9	–	–	11.6–16.5

*CI* confidence interval, *NA* data not available

^a^Date reflects publication date; data collection data not available

^b^New = new case of active TB for that patient; default = defaulted from previous TB treatment regimen; MDR = multidrug resistant TB

**Table 7:** Mean HRQOL scores reported by studies by studies evaluating HRQOL among subjects with post-TB sequelae using home mechanical ventilation (HMV) and other comparison patient groups also on HMV

Instrument	Study (reference number)	Year of data collection	Country	Measures	Patient groups	
					Post-TB sequelae	Other disorders
GHQ–12						
	Dellborg et al. [9, 10, 19, 37]	1992–1995	Sweden	*n* (%)	17 (44)	22 (56)
				Mean total score (95 % CI)	3.5	4.9
	Pehrsson et al. [39]	1994^a^	Sweden	*n* (%)	5 (13)	34 (87)
				Mean total score	4.4	5.1
HAD						
	Pehrsson et al. [39]	1994^a^	Sweden	*n* (%)	5 (13)	34 (87)
				Mean anxiety score	2.3	2.8
				Mean depression score	4	1.8
MACL						
	Dellborg et al. [9, 10, 19, 37]	1992–1995	Sweden	*n* (%)	17 (44)	22 (56)
				Mean total score	2.7	3.3
	Pehrsson et al. [39]	1994^a^	Sweden	*n* (%)	5 (13)	34 (87)
				Range of domain mean scores	3.4–3.6	3.4–3.4
SIP						
	Dellborg et al. [9, 10, 19, 37]	1992–1995	Sweden	*n* (%)	17 (44)	22 (56)
				Mean total score	15	8.3
	Pehrsson et al. [39]	1994^a^	Sweden	*n* (%)	5 (13)	34 (87)
				Mean total score	4.4	5.1
SRI						
	Lopez-Campos et al. [31]	2004–2006	Spain	*n* (%)	12 (10)	103 (90)
				Mean total score	59	58
	Windisch et al. [56, 57]	2003^a^	Germany	*n* (%)	20 (9)	206 (91)
				Mean total score	52	56

*CI* confidence interval

^a^Date reflects publication date; data collection data not available
